# Conceptual Modeling in Systems Biology Fosters Empirical Findings: The mRNA Lifecycle

**DOI:** 10.1371/journal.pone.0000872

**Published:** 2007-09-12

**Authors:** Dov Dori, Mordechai Choder

**Affiliations:** 1 Faculty of Industrial Engineering and Management, Technion, Israel Institute of Technology, Haifa, Israel; 2 Department of Molecular Biotechnology, Rappaport Faculty of Medicine, Technion, Israel Institute of Technology, Haifa, Israel; The Babraham Institute, United Kingdom

## Abstract

One of the main obstacles to understanding complex biological systems is the extent and rapid evolution of information, way beyond the capacity individuals to manage and comprehend. Current modeling approaches and tools lack adequate capacity to model concurrently structure and behavior of biological systems. Here we propose Object-Process Methodology (OPM), a holistic conceptual modeling paradigm, as a means to model both diagrammatically and textually biological systems formally and intuitively at any desired number of levels of detail. OPM combines objects, e.g., proteins, and processes, e.g., transcription, in a way that is simple and easily comprehensible to researchers and scholars. As a case in point, we modeled the yeast mRNA lifecycle. The mRNA lifecycle involves mRNA synthesis in the nucleus, mRNA transport to the cytoplasm, and its subsequent translation and degradation therein. Recent studies have identified specific cytoplasmic foci, termed processing bodies that contain large complexes of mRNAs and decay factors. Our OPM model of this cellular subsystem, presented here, led to the discovery of a new constituent of these complexes, the translation termination factor eRF3. Association of eRF3 with processing bodies is observed after a long-term starvation period. We suggest that OPM can eventually serve as a comprehensive evolvable model of the entire living cell system. The model would serve as a research and communication platform, highlighting unknown and uncertain aspects that can be addressed empirically and updated consequently while maintaining consistency.

## Introduction

Recent years have witnessed unprecedented increases in the number, variety and complexity of information resources available to researchers in the life sciences. We are at a turning point in biological research, where emphasis is shifting from the study of a single molecular process to studying complete cellular pathways and the entire cell as a system. This pivotal time for the life sciences is captured by the words of Kitano: “a *transition is occurring in biology from the molecular level to the system level that promises to revolutionize our understanding of complex biological regulatory systems and to provide major new opportunities for practical application of such knowledge*” [1]. There is now a drive to acquire system-level comprehension of the countless pieces of information that have been gathered thanks to decades of meticulous laboratory research by thousands of scientists. These efforts, many of which are currently considered as contributions to Systems Biology, are aimed at understanding the underlying structure and behavior of biological systems at the molecular, cellular, organism, and habitat levels. Kitano [1] also noted that new tools, ranging from experimental devices to software and analytical methods, are required if we are to meet the challenges of systems biology.

### Overview of the emerging systems biology field

In November 2006, Nature Cell Biology and Nature Reviews Molecular Cell Biology published jointly on the Web (http://www.nature.com/focus/systemsbiologyuserguide) Systems Biology: a User's Guide [2]. This is a collection of Review-type articles concerning the most important approaches and challenges in systems biology. The editors of the guide echo the words of Davidson et al. [3] “*systems biology is essential if we are ever to make sense of biological complexity, as intuitive ‘conceptual’ models quickly reach their limits beyond simple linear pathways*”. Moreover, they affirm: “*The time has come for molecular cell biologists, computer scientists and mathematicians to embrace each other's approaches, as is commonplace in the physical sciences. … More importantly, there is an urgent need to make the next generation of molecular cell biologists ‘systems savvy’. The traditional segregation in higher education of biology from mathematics and physics presents challenges and requires an integration of these subjects for the biologists of the future.*” In the Editorial, systems biology is defined broadly as the integration of complex and highly diverse biological information into a holistic, quantitative and predictive conceptual framework [2]. A key notion here, as in the work of Davidson et al. [3], is the ability of system modeling to be compatible with empirical research, but more specifically, it is suggested that the model must foster empirical predictions. Since systems biology is based on quantitative empirical data and marries informal cartoon-like static flowchart-type models with formal mathematical and computational modeling, it has indeed the promise of generating biological predictions accessible to experimental verification. Moreover, the requirement for modeling and model friendly experimentation to work hand-in-hand, they claim, creates a dynamic interplay that has the potential to result in cyclically better mechanistic understanding and more rigorous interpretation of the system under study. In line with this call to integrate modeling and experimentation, we present a new systems biology modeling approach that has already enabled a significant model-driven experimental finding, which we present as part of this work.

Also in the editorial, after noting how molecular cell biology is emancipating itself from an informal, reductive, hypothesis-driven approach by embracing high-throughput data acquisition, rigorous quantification and mathematical modeling, it is forecast by Kritikou et al. [2] that ultimately a “virtual cell” will be developed. With such an aspiration in mind, we have designed a predictive, conceptual object-process-based model of the mRNA lifecycle. As we demonstrate here, this model facilitates novel insights into this integral cellular subsystem. Moreover, our model potentially represents a new tool for modeling other biological systems.

Adopting a mechanistic view, Davidson et al. [3] contended that traditional biological approaches, which focus on determining the functions of one or a few genes at a time, are not adequate for analyzing large regulatory control systems organized as networks. The need for formal modeling is manifested, among other requirements, by the need to express logical expressions even when describing the expression of a single gene. For example, cis-regulatory elements active within defined spatial limits during development often use AND logic, in that two different transcription factors, each present in a given spatial domain, must be bound to the cis-regulatory DNA at once in order for transcription to be activated. Davidson et al. [3] propose that understanding why a given developmental process occurs requires learning the critical inputs and outputs and their key target sites throughout the genomic regulatory system that controls the dynamic process, and moreover relies on experimental determination of the functional significance of each parameter. In summary, Davidson et al. [3] argue that biological complexity dictates the need for formal modeling, but specifically for models that can work hand-in-hand with empirical research.

### Current approaches to biological modeling

The number of interactions, processes, and transport activities in the living cell is enormous. Therefore, often preceding a quantitative problem of how much or to what extent is the qualitative one of figuring out how and what. Thus, a combined qualitative then quantitative conceptual modeling approach, such as the one adopted in this work, plays a crucial role in facilitating human comprehension of complex cellular mechanisms. Conceptual models advocate the construction of primarily qualitative models, in which biological concepts are put in context with each other in an attempt to gain insights into the function, structure, and dynamics of the biological systems under study. Once a particular, relatively small subsystem in a specific cell location is understood well enough, mathematical tools, such as differential equations, can successfully describe time varying changes. In what follows we survey briefly current approaches and software environments for modeling biological systems, highlighting their advantages and disadvantages.

Modeling efforts in biology are sometimes classified according to their focus on quantitative vs. qualitative aspects. However, oftentimes the two approaches cannot be separated; for example understanding a qualitative process such as the mechanism regulating cell division requires quantitative understanding of this system's dynamics. It must be appreciated that a complex network of protein interactions that influence the activities of cyclin-dependent kinases control major events of the cell cycle, including DNA synthesis, mitosis and cell division. [4] modeled this network using a set of nonlinear differential equations and by numerical simulation predicted its behavior. However, like other researchers before them, they realized that these computer simulations, despite enabling detailed quantitative comparisons between theory and experiment, give little insight into the qualitative dynamics of the control system and do not reveal how molecular interactions determine the fundamental physiological properties of cell replication. To that end, they used bifurcation diagrams as an analytical tool to obtain new views of the dynamic organization of the cell cycle, the role of checkpoints in assuring the integrity of the genome, and the abnormal regulation of cell cycle events in mutants. They validated these insights by analyzing cell cycle regulation in fission yeast. Here, a combined quantitative and qualitative modeling approach is what ultimately provided genuine insights, but this combination is likely paradigmatic.

#### Quantitative Models

One quantitative modeling environment is E-Cell [5], [6], which uses general technologies and theoretical supports for computational biology with the grand aim to allow for precise whole cell simulation at the molecular level. E-cell simulates cell behavior by integrating numerically the differential equations described implicitly by reaction rules. It includes numerical simulations and mathematical analysis technologies to predict, obtain or estimate parameters such as reaction rates and concentrations of molecules in the cell. In spite of all its capabilities, E-cell lacks the ability to specify highly complex systems, based on qualitative data, with multiple components.

Another example of a mathematics-oriented software modeling environment is the Virtual Cell [7], developed for quantitative cell biological research by the National Resource for Cell Analysis and Modeling. Slepchenko [8] described applications of this tool to nucleocytoplasmic transport and intracellular calcium dynamics. The Virtual Cell software environment enables sophisticated quantitative dynamics modeling, such as the one described in [8]. The biological to mathematical mapping allows for separate use of biological and mathematical components, and includes automatic mathematical simplification using pseudo-steady approximations and mass conservation relationships. This mapping allows for direct specification of mathematical problems, performing simulations and analysis on those systems. However, like E-cell, the Virtual Cell software environment lacks the ability to faithfully describe complex systems that are based on qualitative results. It also lacks the capacity to describe multiple interconnected components that need to be modeled at various levels of detail.

Investigating multi-cellular organisms by constructing their conceptual models has been promoted by Harel [9], who also suggested a Turing-like test for biological modeling [10]. Formal modeling of C. elegans development has been carried out by Kam et al. [11] using a scenario-based approach. They have presented preliminary results of a new approach to the formal modeling of biological phenomena based on the language of live sequence charts with the play-in/play-out process. Keet [12] has suggested exploiting existing data better and bringing more structure to the “biological data anarchy” on the Web by enhancing biological information systems with granularity and harnessing Semantic Web technologies.

Some quantitative approaches combine the concept of intelligent computer programs, commonly known as software agents, with mathematical models. Applying an OO and agent-based approach, Webb and White [13] modeled and simulated metabolic and genetic pathways. Due to limitations of the OO paradigm that stem from its origins in the software domain, this model includes such non-biological artifacts as capsules, ports, and connectors that exchange messages, making it less than intuitive.

#### Object-Oriented and UML-based modeling approaches

Conceptual modeling originated with efforts to streamline software development some three decades ago. Therefore, the object-oriented approach, which is the currently accepted paradigm in the software engineering community, has been very popular in recent years for modeling systems in general and biological systems in particular. The object-oriented (OO) approach advocates that objects are the prime entities or building blocks of software systems, and this notion has been recently extended via SysML (www.sysml.org) to systems in general. A basic tenet of OO modeling is the encapsulation principle, which states that objects, the basic building blocks of the model, encapsulate (own) processes, known as methods or operations. The latter do not have their own right of existence as stand-alone things, making it awkward to try to model biological processes. Cell-level biological processes usually involve a host of input, output, and facilitating molecules of all kinds, which are the objects. Since the OO modeling paradigm advocates that each process be a subordinate of some object, an arbitrary choice must be made as to which object is the owner of the process being modeled. This enforced subordination inevitably leads to a counter-intuitive model right from the outset.

BioUML, [14] is an open source software framework for systems biology, which is based on Unified Modeling Language, UML [15], [16], the industry standard in software development. UML caters to the OO paradigm in that its terminology and notation closely follow the notions and capabilities of current OO programming languages.

UML is built on the premise that “Modeling is the designing of software applications before coding” [17]. UML-based modeling approaches like BioUML are object-oriented, meaning that their main building blocks are objects, which are primary static entities that own processes. The inherent orientation of UML towards software, its unnecessary complexity [18] and its model multiplicity problems [19] cause intra- and inter-model consistency problems [20].

In general, the current Object-Oriented approach to modeling and developing software systems is not suitable for representing effectively biological concepts, because, as argued, it cannot model concurrently in a single type of diagram both the objects, e.g., a protein, and the processes, e.g., transcription, that transform (create, consume, or change the state of) these objects. UML 2.0 [16], for example, includes 13 different types of models, each with its own diagram type, separate set of symbols and concepts. Moreover, the lack of the process as a stand-alone concept in the OO modeling approach is a major hindrance for modeling biological systems, which are mostly process-intensive. Finally, many software engineers find it difficult to master the UML modeling framework, making it unrealistic to expect biologists to employ it in a valuable way to model biological systems.

Systems Biology Markup Language, SBML [21] is an open, XML-based format for representing biochemical reaction networks and describing models common to research in many areas of computational biology, including cell signaling pathways, metabolic pathways, and gene regulation. SBML data objects use a graphical notation based upon UML, which in turn is translated into XML. CellML [22] is another XML-based language for storage, sharing, exchange, and reuse of computer-based mathematical models. It includes information about model structure, equations describing processes, and metadata to search for model components.

The recent Systems Modeling Language, SysML [23] initiative offers no solutions to the problems of current OO approaches to modeling, as it is based on UML and therefore suffers from most of UML's deficiencies, namely multiple diagram types and segregation between structure and behavior.

#### Specialized modeling frameworks

Kohn [24] has proposed a graphical method for mapping bioregulatory networks and representing multimolecular complexes, protein modifications, and actions at cell membranes and between protein domains. The symbol conventions, defined for these molecular interaction maps, accommodate multiprotein assemblies and protein modifications and thus can generate combinatorially large numbers of molecular species. However, most of the 20 or so pictogram symbols are highly specialized. For example, one of them is defined as “Transport of Protein A from cytosol to nucleus”. Clearly, this method is limited to modeling very specific systems.

In general, when considering human-readable diagrammatic representations, it is notable that the current informal ways most biologists draw diagrams means that correct biological interpretation depends entirely on the reader's knowledge [25]. Kitano et al. [26] recount examples where an arrow symbol has four different potential interpretations and indicate rightly that such ambiguities become a major problem as the size and complexity of the system increases, highlighting the need for formality to avoid ambiguity. Process diagrams proposed by [25] that make use of CellDesigner [26] are state transition-based.

Using different arrowhead shapes CellDesigner diagrams focus on conveying the semantics of several process types prevalent in signaling, such as translocation, catalysis, splitting, phosphorylation, or state transition. Formalized process diagrams have been used to describe signal-transduction cascades and pathway maps, and are readable and precise as long as the network is not too large. However, scalability is an issue. There is no way to refine mechanisms and designating new pictograms for each new reaction type is problematic. Moreover, as Blinov et al. [27] observe, because process diagrams require explicit representation of all the species (which in our ontology are referred to as objects) at some level, they omit the vast majority of species and reactions, which are processes in our ontology, that could potentially be generated during signaling.

In an attempt to solve this problem, Blivnov et al. have introduced graphical rules to allow the connectivity of proteins in a complex to be represented explicitly. These rules provide a means to visualize comprehensibly protein-protein interactions. Nevertheless, for more general biological modeling, process maps are likely to be of unmanageable size due to combinatorial complexity.

An example of a combined quantitative-qualitative model is the work of Tyson et al. [28], who modeled the dynamics of cell cycle regulation. They applied a systems dynamics-based approach and bifurcation diagrams to provide a new perspective on cell cycle checkpoints and mutant phenotypes in fission yeast. In this model, qualitative changes can occur, for example, when a stable steady state loses its stability or even ceases to exist and is replaced by an oscillatory solution. Such an event is in the nature of the recurrent solutions of a dynamic system and is necessary for a model attempting to characterize the cell cycle. These qualitative changes, called bifurcations, happen at specific values of the parameters termed bifurcation points and are described by a one-parameter bifurcation diagram. These are two-dimensional graphs, where each axis shows some quantity and the quantitative analysis yields certain meaningful qualitative results, such as the G1, G2, or metaphase checkpoints in the fission yeast cell cycle.

BioTapestry [29] is the latest interactive tool for building, visualizing, and simulating genetic regulatory networks. It is designed around the concept of a developmental network model, intended to handle large scale models and represent systems that exhibit increasing complexity over time. The system supports data generated by perturbing the expression of specific genes, portrays views of the network during development, and lays out network models.

#### Problems with current models

The current state of affairs, reflected in this survey, is that there are quite a number of modeling approaches and software environments for modeling biological systems, but many of them are object-oriented, hindering direct and explicit process modeling, which is at the heart of systems biology. Moreover, since most approaches are non-scalable and specialized for specific types of cellular reactions or subsystems, they cannot be extended naturally to modeling the entire cell, not to mention organisms, societies, habitats and ecologies.

Perhaps most importantly, according to the definition of systems biology, models are supposed to advance research, yet none of the existing modeling approaches or systems have been shown to promote innovative questions that trigger experiments to confirm or refute assumptions emerging from the model. Such a disappointing situation indicates that a totally different modeling approach is in order. This situation was a major stimulus for the work presented here, a non-traditional conceptual modeling approach that has already stimulated a new empirical finding concerning the mRNA lifecycle.

#### Our approach to biological modeling

Representing the vast amount of ever increasing knowledge formally, yet accessibly, can be compared to putting the pieces of a gigantic puzzle together, mandating adoption of a common evolving grand model. The model needs to be founded on a compact generic set of the most basic ontological building blocks in order for it to be general enough to serve as a basis for modeling the gamut of biological systems, from molecules to ecosystems.

We submit that stateful objects and processes that transform them, along with several types of links, as advocated by OPM—Object-Process Methodology [30], constitute a mandatory and sufficient set of ontological building blocks to enable conceptual modeling of biological systems with various scales and complexities. Moreover, such building blocks allow reasoning that links theory with empiricism. We envision an OPM-based comprehensive shared Web-accessible modeling framework of the entire cell, which, if and when created, would enable the evolution of state-of-the-art knowledge in biology. This model will keep pace with the rapid evolution of knowledge, and will be revised constantly and updated with new findings and conjectures.

In the present study we aimed to carry out a modest first step toward this admittedly ambitious goal. As the yeast mRNA life cycle is a key cellular system, we chose it to be our case in point for conceptual modeling that employs Object-Process Methodology. In the next section we explain in more detail why we chose Object-Process Methodology, OPM.

#### Object-Process Methodology

Object-Process Methodology, OPM [30] is a holistic approach to the study and development of complex systems that caters to human intuition while maintaining a formal framework. The living cell is a prime example of a highly complex system, in which the two main system aspects—structure and behavior—are highly intertwined and hard to separate. Motivated by the requirement of a single model to represent these two major system aspects, OPM is founded upon two elementary building blocks—objects and processes—which represent concurrently the system's structure, i.e., the objects, or components, that comprise the system, and behavior, i.e., the processes that transform the system's objects by creating them, consuming them, or changing their states, in a balanced way without highlighting one at the expense of the other.

The elements of OPM ontology are entities and links. A complete list of OPM elements with their symbols and definitions is provided in Table S1. Entities, the basic building blocks of any system modeled using OPM, are of three types: *objects*, possibly with *states* (stateful objects), and *processes*. An object is a thing that exists, possibly in some state, while a process is a thing that can transform objects. More specifically, a process is a thing that transforms objects, namely creates one or more objects, consumes one or more objects, or changes the state of one or more objects. Examples of biological objects are Protein, Cell, and Organism, and examples of biological processes are Cleavage, Mitosis, and Birth.

A link can be structural or procedural. A structural link expresses a static, time-independent relation between pairs of entities. The four fundamental structural relations are: aggregation-participation, generalization-specialization, exhibition-characterization, and classification-instantiation. An example of using the aggregation-participation structural relation is derived from the phrase “The eukaryotic cytoskeleton is composed of microfilaments, intermediate filaments and microtubules.” (http://en.wikipedia.org/wiki/Cell_(biology)#Subcellular_components) In OPM, this statement is interpreted such that the Eukaryotic Cytoskeleton is the aggregating object, the whole, which consists of the three objects which are parts of the Eukaryotic Cytoskeleton, each being a set of objects: the Microfilaments Set, the Intermediate Filaments Set, and the Microtubules Set. Unidirectional and bidirectional tagged structural links enable creation of additional user-defined links with specified semantics. A procedural link connects entities (objects, processes, and states) to describe the behavior of a system. The behavior is manifested in three major ways: (1) a process can *transform* (generate, consume, or change the state of) one or more objects; (2) an object can *enable* one or more processes without being transformed by them, in which case it acts as an *enabler*, i.e., a human *agent* or an inanimate *instrument*; and (3) entities can *trigger events* that invoke processes if some conditions are met. Accordingly, a procedural link can be a transformation link, an enabling link, or an event link. A *transformation link* expresses object transformation, i.e., object consumption, generation, or state change. An *enabling link* expresses the need for a (possibly state-specified) object to be present in order for the enabled process to occur. The enabled process does not transform the enabling object. An *event link* connects a triggering entity (object, process, or state) with a process that it invokes.

The Gene-Ontology (GO) [31] project sets out to provide a defined, universal vocabulary for describing gene and gene product attributes in any organism. The three organizing principles of GO are cellular component, biological process, and molecular function. A comparison between GO principles and OPM entities is useful as it emphasizes the advantages of OPM ontology. A cellular component corresponds to an OPM object and a biological process corresponds to an OPM process. However, molecular function does not have a clear OPM equivalent. According to the definition of process in [31], a biological process is a “series of events accomplished by one or more ordered assemblies of molecular functions.” Examples include signal transduction and alpha-glucoside transport. A GO molecular function “describes activities, such as catalytic or binding activities, that occur at the molecular level.” Examples include catalytic activity, binding, or adenylate cyclase activity. Problems with this definition for molecular function are admitted in [31], “It can be difficult to distinguish between a biological process and a molecular function, but the general rule is that a process must have more than one distinct step.” We contend that any ontology that lacks precise, clear-cut definitions, and relies on examples as part of the definition, is problematic. What is the meaning of step? Is it identical to function, and if so, is function in turn identical to activity? If so–why use so many terms, and if not, how do they differ? Indeed, it is unclear how to differentiate between GO functions and GO processes. OPM takes another approach where it does not distinguish between simple and complex processes, just as it does not distinguish between simple and complex objects. For example the GO molecular function “pre-mRNA 3′-splice site binding” (taken from the actual GO file) in OPM ontology is the OPM process of binding of the cellular component (OPM object) “pre-mRNA 3′-splice site”, which changes that object from state unbound to state bound. We advocate that the OPM ontology is more precise and intuitive.

Two semantically equivalent modalities, one graphic and the other textual, are used to describe each OPM model. A set of inter-related Object-Process Diagrams (OPDs), showing portions of the system at various levels of detail, constitute the graphical, visual OPM formalism. Each OPM element is denoted by a symbol in an OPD, and the OPD syntax specifies correct and consistent ways by which entities can be connected via structural and procedural links, such that each legal entity-link-entity combination bears specific, unambiguous semantics. OPCAT [32] is a software environment that supports OPM-based system modeling and evolution.

The Object-Process Language (OPL), which is the textual counterpart of the graphical OPD, is a dual-purpose language, oriented towards humans as well as machines. Catering to human needs, OPL is designed as a subset of English, which serves domain experts (e.g., biologists) and system architects, engaged jointly in modeling a complex system. Every OPD construct is expressed by a semantically equivalent OPL sentence or phrase. According to the modality principle of the cognitive theory of multimodal learning [33], this dual graphic/textual representation of the OPM model increases the human processing capability. Indeed, it has been our experience that human understanding of the OPM model is enhanced by the convenient opportunity of reflecting upon both the graphic and textual model representations, whereby what is missed in one modality can be grasped when considering the other one.

**Figure 1:**
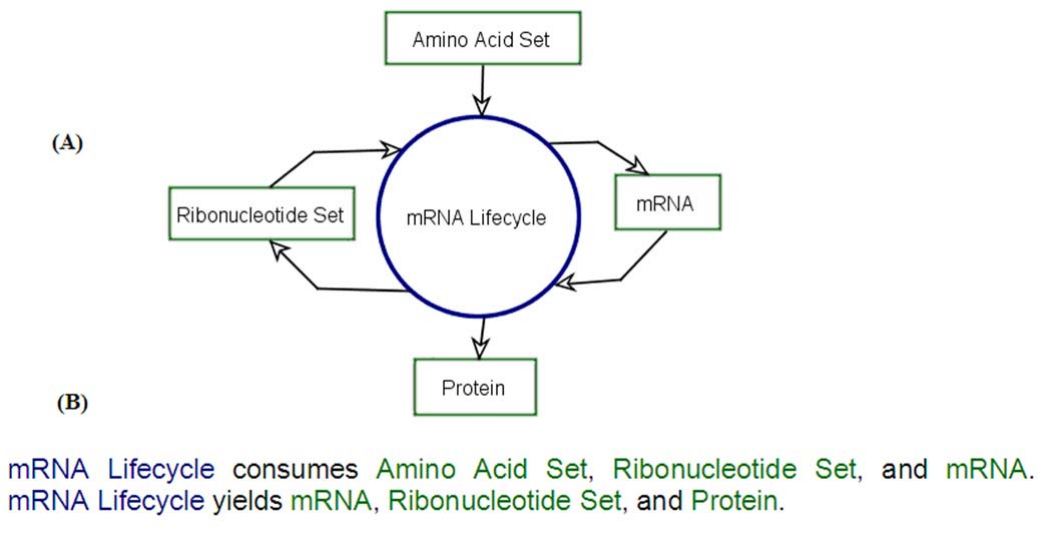
A top-level view of the mRNA Lifecycle OPM model. (A) An Object-Process Diagram (OPD) showing the top-level, bird's eye view of the process mRNA Lifecycle, which generates the object Protein from the object Amino Acid Set. The process, marked as the blue ellipse, shows the generation of mRNA from Ribonucleotide Set and the degradation of mRNA back to Ribonucleotide Set. (B) The corresponding Object-Process Language (OPL) text that was generated automatically by OPCAT, the software that supports OPM modeling.

Figure 1(A) is an Object-Process Diagram (OPD), showing the top level, bird's eye view of the process mRNA Lifecycle, which generates the object Protein from the object Amino Acid Set. Figure 1(B) lists the corresponding two OPL sentences that were generated automatically by OPCAT:

mRNA Lifecycle consumes Amino Acid Set, Ribonucleotide Set, and mRNA.

mRNA Lifecycle yields mRNA, Ribonucleotide Set, and Protein.

Examining these sentences, we see that each arrow from an object to the process (mRNA Lifecycle) has the semantics of consumption (degradation, or destruction), while each arrow from the process to an object has the semantics of result (creation, or generation). During the process mRNA Lifecycle, Ribonucleotide Set and mRNA are both consumed and generated, while Amino Acid Set is consumed to create Protein.

#### Complexity Management

A major problem with most graphic modeling approaches is their scalability. As the system's complexity increases, the graphic model becomes cluttered with symbols and their connecting links. The limited channel capacity [33] is a cognitive principle which states that there is an upper limit on the amount of detail a human can process before being overwhelmed. This principle is addressed by OPM and implemented by OPCAT via three abstraction/refinement mechanisms. These enable complexity management by providing for the creation of a set of interrelated OPDs (along with their corresponding OPL paragraphs) that are limited in size, thereby avoiding information overload and enabling comfortable human cognitive processing. The three refinement/abstraction mechanisms are: (1) *unfolding/folding*, which is used for refining/abstracting the structural hierarchy of a thing and is applied by default to objects; (2) *in-zooming/out-zooming*, which exposes/hides the inner details of a thing within its frame and is applied primarily to processes; and (3) *state expressing/suppressing*, which exposes/hides the states of an object.

**Figure 2:**
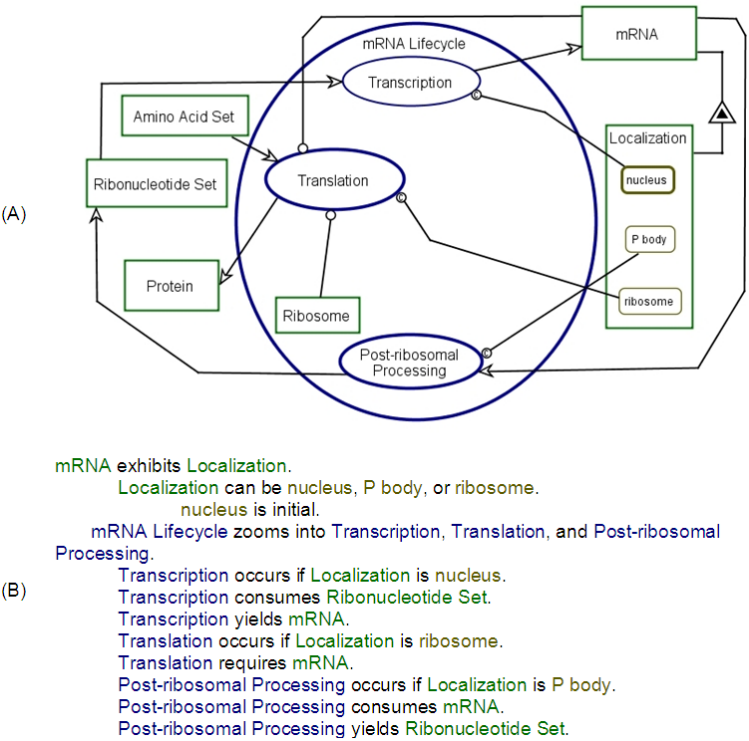
Zooming into the mRNA Lifecycle process from Figure 1. (A) An OPD in which the mRNA Lifecycle process from Figure 1 is in-zoomed into the three main subprocesses Transcription, Translation, and Post-ribosomal Processing. Time in an OPD flows from top to bottom, so Transcription is the initial process, Translation comes next, and finally Post-ribosomal Processing. While more detailed, the view of the system in this figure is fully consistent with its more abstract ancestor, shown in Figure 1. For the sake of simplicity transport-related processes are not depicted here yet. mRNA exhibits the attribute Localization, which can be at the states ribosome, nucleus, or P body. This relation between mRNA and its Localization attribute is denoted by the black-in-white triangle connecting these two objects. A thick process contour indicates that this process is in-zoomed in a different, more detailed OPD to show the inner content of that process. For example, the Translation process is zoomed in Figure 4. (B) The OPL text that explains the graphical model. For example, the OPL sentence Transcription occurs if Localization is nucleus. is denoted in the OPD by the link from the nucleus Localization to the Transcription process. In OPM, this link is called condition link, and it is denoted by the letter ‘c’ inside the link's circle end. In free text this means that transcription takes place in the nucleus.

Figure 2 provides examples of these three abstraction/refinement mechanisms. The mRNA Lifecycle process from Figure 1 is in-zoomed, exposing three main subprocesses: Transcription, Translation, and Post-Ribosomal Processing. Two examples of unfolding are shown in Figure 2. One is the unfolding of mRNA Polymerase II as *part* of Nucleus. The other is the unfolding of Localization as an *attribute* of mRNA. Finally, state expression is exemplified when the possible states of Localization—nucleus, P body, and ribosome—are expressed. Using flexible combinations of these abstraction/refinement mechanisms, OPM enables a system to be specified to any desired level of detail, without losing legibility or comprehension of the specification. The complete OPM system specification is expressed graphically by the resulting set of consistent, inter-related OPDs, and textually by the corresponding OPL paragraphs.

#### The mRNA Lifecycle

The expression of protein encoding genes is a complex process that determines which genes are expressed as proteins at any given time, as well as the relative levels of these proteins [34]. This process involves several distinct stages, (i) RNA synthesis, or transcription, (ii) RNA processing (after processing is completed, the RNA is considered mRNA), (iii) mRNA transport from the nucleus to the cytoplasm (in eukaryotes), (iv) protein synthesis, or translation, (v) mRNA degradation, and (vi) posttranslational modifications (including degradation) of the proteins. In eukaryotes, transcription is carried out by three different RNA polymerases, each responsible for transcribing a distinct class of RNAs. RNA polymerase II (pol II) is responsible for transcribing protein-encoding RNAs, namely mRNAs, which are the focus of this paper. Work from many laboratories has established that regulation of the aforementioned stages of pol II-mediated gene expression is coordinated [35]. Thus, in order to understand the expression of protein-encoding genes we need to consider the entire multi-stage process, as each stage can be regarded as a subdivision of a continuous gene expression process. Correctly and accurately specifying this complex process is a formidable task, beyond the realms of free text. Clearly, it calls for the use of an appropriate modeling language and methodology.

Transcription by pol II, the first stage in the expression of protein-encoding genes, produces RNA–the primary transcript. This primary transcript is processed to yield an mRNA (usually shorter than the primary transcript) that contains a 5′ cap (m(7)GpppN) and 3′ poly(A) tail. These two tags are critical for the appropriate function, localization and stability of the mRNA [34]. Following its synthesis in the nucleus, the mRNA is transported to the cytoplasm, where it is recognized by ribosome(s)-the protein synthesis machinery. The mRNA is then used as a template for translation into protein, the amino acid sequence of which is related to the nucleotide sequence of the mRNA [34]. The last stage of the mRNA lifecycle in the cytoplasm is its decay, which is carried out by an array of decay factors [36], [37]. Each one of the stages described above is tightly regulated. Once produced, the mRNA is the key target in the regulation of the gene expression, referred to as post-transcriptional regulation. One aspect of post-transcriptional regulation is at the level of mRNA localization within the cell, for example nuclear vs. cytoplasmic localization.

Recently, a new venue for mRNA localization was uncovered that revolutionized our view of how gene expression is regulated post-transcriptionally. Specifically, yeast mRNA can be localized in discrete cytoplasmic foci together with a number of mRNA decay factors and limited repertoire of translation factors, mostly translation repressors. These foci, termed processing bodies (P bodies), represent complexes where mRNA degradation can occur, since mRNA decay intermediates [38] and many factors of the major mRNA decay pathway reside in P bodies [38]–[49]. The discovery of P bodies in yeast and related bodies in higher eukaryotes, e.g., dcp bodies, GW bodies, brings the spatial control of macromolecule to the focus of our attention.

A different kind of cytoplasmic foci, referred to as stress granules, have been discovered in higher eukaryotes under various stress conditions (recently reviewed in [49], [50]). Unlike P bodies, these foci contain several translation factors, but not the ribosomal large subunit [51], [52]. Stress granules are believed to be sites where non-translating mRNA resides during stress conditions [53]. Association of mRNAs with stress granules was proposed to represent a mechanism for translational repression. This kind of repression can be reversible, as the mRNA can be transported back to the ribosome when conditions favor translation. Anderson and Kedersha [54] proposed that mRNAs in stress granules are subjected to triage: first they are monitored for integrity and composition, and then they are sorted for productive translational initiation or targeted to degradation. In addition, it has been suggested that stress granules may communicate with P bodies when sorting the mRNA for degradation [55]. In yeast, the organism under study here, no stress granules have been identified and the bulk of known P bodies do not contain translation factors [43]. Recently, work from Parker's group has revealed that mRNAs in the yeast P bodies are not necessarily degraded, but rather can be transported to the ribosome for translation [56]. Thus, yeast P bodies may have dual function, carrying out the functions of both mammalian P bodies and stress granules.

Most published works to date have used fluorescent microscopy to study P bodies or stress granules. This technology allows the detection of large complexes whose fluorescence is above that of the background, but small P bodies might escape detection. Interestingly, though, Aragon et al. [57] have recently reported that mRNAs in starved yeast cells are sequestered in proteinatious complexes and therefore resist standard extraction procedures. These mRNAs could be recovered by disrupting the complexes with proteases. The released mRNA can then be analyzed using whole genome technology. The authors proposed a plausible model in which the protecting complexes are P bodies [57]. If proved correct, the differential mRNA extraction technique will allow analyzing even small P bodies and also obtain, for the first time, quantitative results at the organism level.

It has been contended both for yeast [38], [54], [55] and higher eukaryotes [48], [56] that there is frequent shuttling of mRNA between the ribosome (or poly-ribosome) and these cytoplasmic bodies. Moreover, it has been suggested that two of the cytoplasmic structures, i.e., the ribosome, which activates translation, and the P body, which represses it, compete for mRNA. The outcome of this competition determines mRNA translatability and hence protein synthesis [58]. To demonstrate shuttling of mRNA between these two complexes, investigators utilized specific drugs. Drugs that block mRNAs within the ribosomes cause P bodies to disassemble, whereas drugs or mutations that compromise mRNA loading onto the ribosomes enhance the assembly of P bodies [38], [48], [54], [50], [59]. Such studies reveal that P bodies are dynamic structures, for their mass varies as the mRNA is transported back and forth between P bodies and ribosomes. It has also become clear that the balance between these two sub-cytoplasmic compartments, and hence the P bodies' size, is responsive to environmental signals [43], [48], [54], [58]. Thus, external signals, such as starvation, UV irradiation, or changes in osmolarity, can trigger mRNA redistribution between the two compartments. It was the complexity and intricacy of processes constituting the mRNA lifecycle, outlined above, which triggered our realization that conceptual modeling might likely contribute to understanding this particular cellular sub-system. Moreover, we anticipated that the modeling should raise empirically addressable questions, the answers to which would help researchers comprehend better mRNA biology. Indeed, the modeling activity has raised at least one research question, the localization of eRF3 in P bodies, which we have confirmed experimentally.

## Methods

OPM allows us to model the system under study—the mRNA lifecycle—at various hierarchically arranged levels of detail. We started modeling only established knowledge concerning the mRNA lifecycle. Figure 1A is the System Diagram (SD). It is the top level Object-Process Diagram (OPD), which provides a bird's eye view of the system, illustrating graphically the mRNA Lifecycle system in a nutshell. In the mRNA Lifecycle process, Ribonucletides are consumed to generate mRNA. mRNA is produced by the Synthesis process, which, as the model's next levels of detail reveals, includes Transcription and Nucleo-cytoplasmic Transport. The main product of the mRNA Lifecycle process is Protein, which is synthesized during the Translation process. Stages that follow Translation take place in the P bodies and might produce Ribonucletides, as detailed below. To complete the lifecycle, one option that occurs following translation (see below) is that the mRNA is decomposed back into its constituent Ribonucletides.

Figure 1(B) is the automatically generated textual description, called Object-Process Language (OPL), expressing what the Object-Process Diagram (OPD) shown in Figure 1(A) tells us graphically. This OPL paragraph, a collection of OPL sentences, is equivalent in its informational content to its corresponding OPD.

## Results

Figure 2(A) shows an OPD in which the mRNA Lifecycle process from Figure 1 is zoomed in to expose its three main sub-processes, Transcription, Translation, and Post-ribosomal Processing. This enables us to visualize the details of the mRNA Lifecycle process. The convention is that within an in-zoomed process, there is a timeline—the Y axis of the diagram—that flows from the top of the in-zoomed process ellipse to its bottom. Accordingly, Transcription happens first, followed by Translation, and finally the Post-ribosomal Processing takes place. Parallel or alternative processes are depicted at the same height. Cycles and loops are easily expressed, and the mRNA cycle as a whole is an example. As discussed in the introduction, an important feature of the mRNA Lifecycle is the Localization of the mRNA, which we model as an attribute of mRNA (indicated by the black-inside-white triangle link), with each Localization state being a concrete localization. It is common knowledge that mRNA is produced in the nucleus and then transported to the cytoplasm, where it is translated and degraded [34].

In the cytoplasm, mRNA can be located in various complexes, such as the ribosome or P body. It is quite possible that the yeast cytoplasm contains other large bodies that accommodate mRNA. However, as such bodies have not been reported, the only two cytoplasmic mRNA locations in our model are the ribosome (including also poly-ribosome) and P bodies. It has not been established whether mRNA can move in the cytoplasmic matrix, unattached to any complex. Trying to include this option in our initial model rendered the model more complex with no tangible benefit. Following Occum's Razor, we therefore assumed the simplest option, i.e., that mRNA does not reside in the cytoplasmic matrix as an unbound molecule. Thus, Figure 1(A) shows only three mRNA Localization states: nucleus, P body, and ribosome. Interestingly, when only these three mRNA Localization states are considered, a question arises regarding what is the first cytoplasmic location of mRNA after it is transported from the nucleus.

The model leaves only two options: either ribosome or P body. Although ribosomes have been implicated as the mRNA acceptor in the cytoplasm [59], an as yet unconsidered possibility raised by this model is that in some cases a P body can be the first cytoplasmic location for mRNA. Indeed, Jean Marx has noted that P bodies are found around the nucleus [60]. The conjecture that P bodies may be the first cytoplasmic location for mRNA illustrates the utility and the value of a formal, expressive conceptual modeling approach in provoking new ideas and viewpoints. As we show below, our modeling framework indeed triggered an experiment that provided new, significant information related to the mRNA lifecycle.

**Figure 3:**
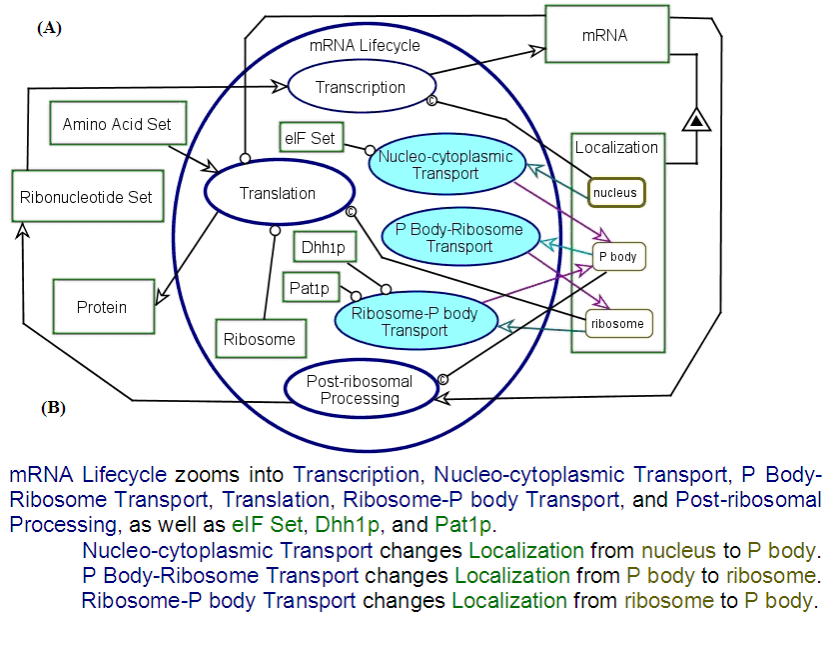
Adding the mRNA transport processes. (A) Three mRNA transport processes, marked in cyan, have been added to the OPD of Figure 2: Nucleo-cytoplasmic Transport, P Body-Ribosome Transport, and Ribosome-P body Transport. According to our model, the cytoplasmic mRNA is localized either in the ribosome or in the P bodies. For simplicity, possible transport from the nucleus to the ribosome is not modeled here. Therefore, the Nucleo-cytoplasmic Transport changes the Localization of the mRNA from nucleus to P body. The two other transport processes are the inverse of each other: P Body-Ribosome Transport, which changes the Localization of the mRNA from P body to ribosome, while Ribosome-P body Transport, which does the opposite. (B) The sentences of the OPL paragraph listing the processes and objects into which mRNA Lifecycle zooms and how each transport process changes the value of the Localization attribute of mRNA between nucleus, P body, and ribosome.

Figure 3 shows a refinement of the mRNA lifecycle OPM model of Figure 2, in which three mRNA Transport processes, marked in cyan, have been added: Nucleo-cytoplasmic Transport, P Body-Ribosome Transport, and Ribosome-P body Transport. According to our model, the cytoplasmic mRNA is localized either in the Ribosome or in the P body. The Nucleo-cytoplasmic Transport, which requires three factors, eIF Set, Dhh1p, and Pat1p, changes the Localization of the mRNA from nucleus to P body. A pair of input/output links, the cyan and purple arrows, denote this transport. The two other transport processes are the inverse of each other: P Body-Ribosome Transport changes the Localization of the mRNA from P body to ribosome and induces translation initiation, while Ribosome-P body Transport does the opposite, repressing translation. As in the Nucleo-cytoplasmic Transport, for each of these two transport processes there is a pair of input/output links, the cyan and purple arrows. If we follow the sequence of these alternating input/output links (cyan and purple arrows), we get that the Localization of mRNA, which starts at the nucleus, changes to P body and then to ribosome. This can be followed by alternating between ribosome and P body, possibly many times, until the mRNA is consumed (degraded) by the Post-ribosomal Processing, as indicated by the consumption link—the arrow in Figure 3 from mRNA to that process. As discussed in the Introduction, dynamic transport of mRNAs between the two compartments has been proposed, but little is known about the transport mechanism, in particular, whether it is direct or involves the cytoplasmic matrix. This black box is yet another example of a model-triggered potential area of research. Two mRNA Decay Factors, Pat1p and Dhh1p, are implicated in the transport of mRNA from the ribosome to the P bodies [54]. These two factors are therefore depicted as instruments that enable the Ribosome-P body Transport process.

**Figure 4:**
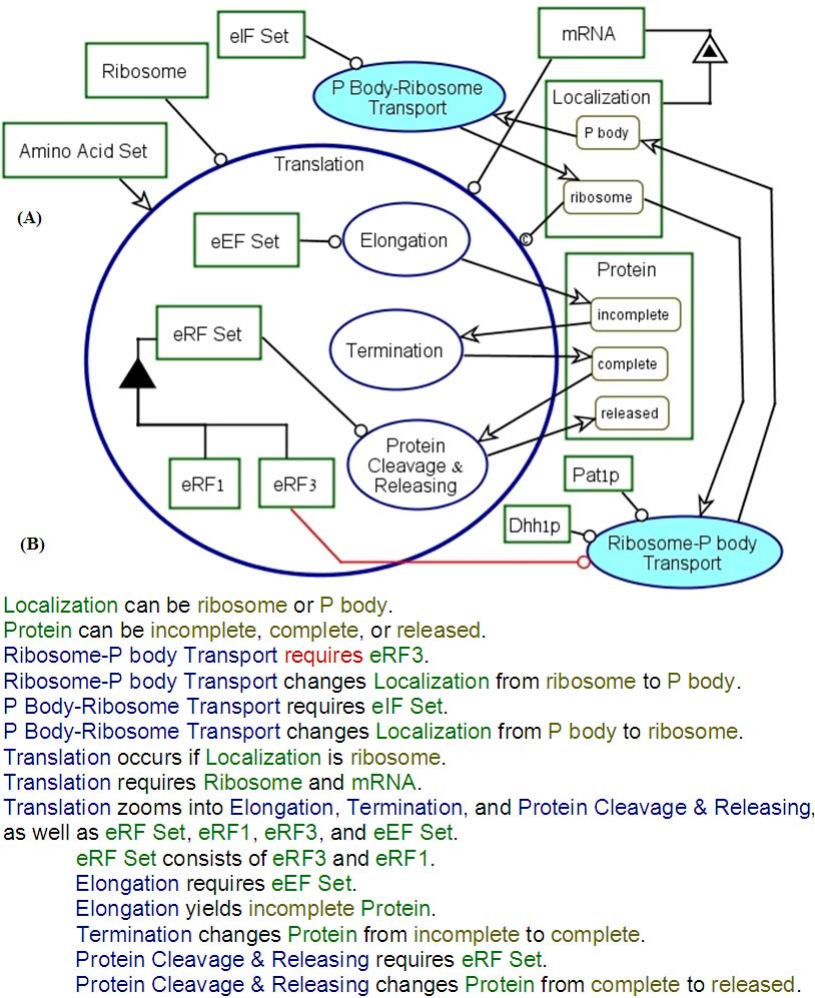
Zooming into the Translation process. (A) Zooming into the Translation process exposes its three subprocesses that follow P Body-Ribosome Transport (initiation): Elongation, Termination, and Protein Cleavage&Releasing. The corresponding factors involved as instruments in these processes are also shown linked to the processes with an instrument link (a line ending with a circle at the process end). The object eEF Set is the instrument for the Elongation process, while eRF Set with its members, the factors eRF1 and eRF3, is the instrument for the Protein Cleavage&Releasing process. Our conjecture is that eRF3 is the factor which is also involved as instrument for the Ribosome-P body Transport process. Since there is no proof for this as yet, the instrument link from eRF3 to Ribosome-P body Transport is colored red, denoting uncertainty. (B) The OPL text of the OPD in (A). Note that the word requires in the OPL sentence Ribosome-P body Transport requires eRF3. denotes the same uncertainty regarding the role of eRF3 as instrument to the Ribosome-P body Transport process, analogous to the instrument link from eRF3 to Ribosome-P body Transport in (A).

In Figure 4, the Translation process is in-zoomed, exposing its three subprocesses: Elongation, Termination, and Protein Cleavage&Releasing, which are executed in this order. Translation initiation is depicted in Figure 3 as P Body-Ribosome Transport. The corresponding factors involved as instruments in these processes are also shown linked to the processes with an instrument link (a line ending with a circle at the process end). The object eEF Set is the instrument for the Elongation process, while eRF Set with its members, the factors eRF1 and eRF3, is the instrument for the Protein Cleavage&Releasing process.

eRF3 is an instrument for translation termination (see [61] and references therein), which has also been shown to be involved in mRNA decay [62]–[64]. These observations provoked us to propose that eRF3 helps coupling between translation termination and P body assembly by serving as an instrument for the Ribosome-P body Transport process. Our model predicts that eRF3 is transported together with the mRNA to P bodies. Indeed, eRF3 is associated with mRNP [63], [64].

**Figure 5:**
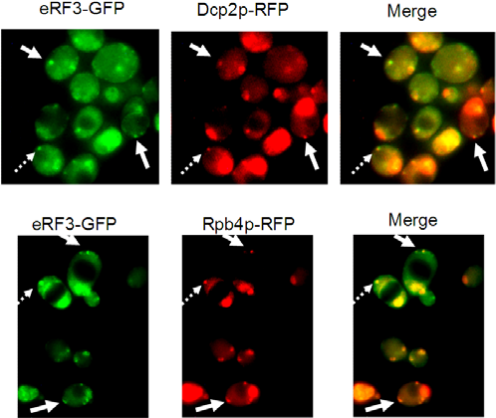
Colocalization of eRF3-GFP with P bodies markers Dcp2p and Rpb4p. Some P bodies are indicated by arrows (different kinds of arrows point at different P bodies). P bodies were examined after 7 days starvation in stationary phase as described in (Lotan et al., 2005). Merging of the green (eRF3-GFP) and the red (Dcp2p-RFP or Rpb4p-RFP, as indicated) channels was done using PhotoShop software. Note that if the green and red foci colocalize the resulting foci are yellow.

This prediction prompted us to examine whether eRF3 is a component of P bodies. Figure 5 demonstrates that eRF3 can indeed be found in cytoplasmic foci of starved cells, together with two known P body constituents. It should be emphasized that association of eRF3 with P bodies was observed after long-term starvation in a stationary phase. We surmise that localization of eRF3 with P bodies is a special case that is subject to specific regulation. This finding is a direct consequence of the conceptual modeling process we engaged in and a conjecture triggered by the OPM model of the mRNA lifecycle that was confirmed experimentally.

**Figure 6:**
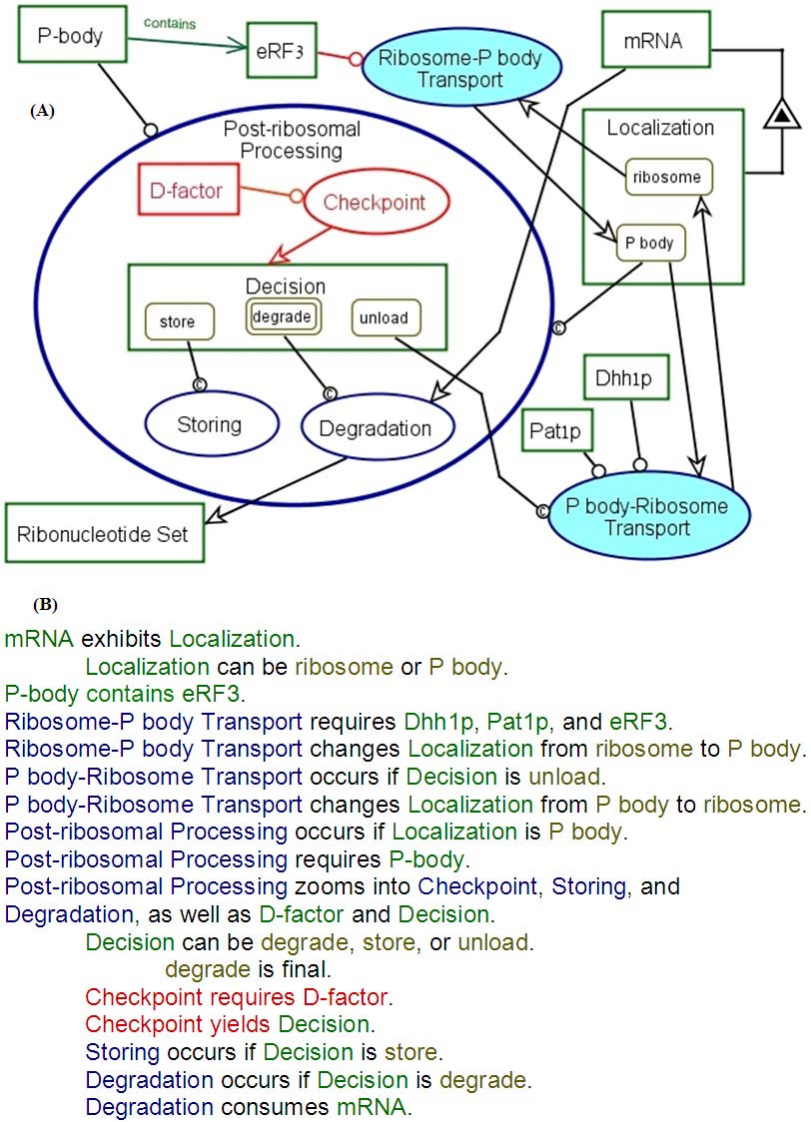
Zooming into Post-ribosomal Processing. (A) Zooming into Post-ribosomal Processing exposes its subprocesses Checkpoint, Storing, and Degradation, as well as the objects D-factor and Decision. As before, red indicates uncertainty or hypothesis: We propose that D-factor is the instrument for the process we call Checkpoint, which in turn, determines whether to store, degrade, or unload the mRNA for reuse. Green links denote an uncertain conjecture that was confirmed in this work by our experiments. Here, the structural link from P Body to eRF3 and the tag contains along it are green, denoting that we demonstrated experimentally our model-based conjecture that P-body contains eRF3. (B) The OPL text of the OPD in (A). Note the red color of the words in the sentences Checkpoint requires D-factor. and in Checkpoint yields Decision. The red denotes that we are not certain whether Checkpoint and D-factor exist, and if so whether Checkpoint requires D-factor. On the other hand, the green color of the word contains in the sentence P-body contains eRF3 indicates our success at experimentally proving our model-based conjecture that P-body contains eRF3.

Continuing our OPM-based conceptual modeling process, Post-ribosomal Processing from Figure 3, which is the final process of the mRNA Lifecycle, is in-zoomed in Figure 6, showing the subprocesses that the mRNA undergoes within the P body. As indicated in the introduction, the fate of the mRNA in the P body is complex, as it can be degraded, stored, or unloaded. Our model therefore includes a regulated decision-making process, called Checkpoint, which controls execution of one of the following options: (1) degrade (which includes decapping as a first subprocess), (2) store (sequestrate), and (3) unload (a condition for the P body-Ribosome Transport process, which releases mRNA back to the Ribosome). Accordingly, the Object-Process Diagram in Figure 6 specifies that Decision can be in one of the three states: degrade, which activates the Degradation process, store, which sequestrates the mRNA, or unload, which activates the P body-Ribosome Transport, the process that initiates translation. The red color of Checkpoint indicates that it is a hypothetical process. We propose that the object D-factor, as yet a hypothetical factor, is the instrument that governs the Checkpoint process. This process generates the logical object Decision, whose state determines which one of the above three options will be executed. D-Factor, also colored red, as its identity remains to be determined, could be any one of the known P body constituents. Other options include kinase, phosphatase, ubiquitin E3 ligase, acetylase, protease, RNase, chaperon, etc. It is likely, although not necessary, that it resides in the P body.

### Generalizing the opm-based modeling process

Having presented the modeling process for specific portions of the mRNA lifecycle in some detail, we now outline general guidelines for OPM-based conceptual modeling of a biological system. At the molecular level, all biological systems obey relatively few common rules. Molecules can interact with other molecules, change their molecular environment if they have enzymatic activity, or affect localization within the cell. All these activities are included in our mRNA lifecycle model, which can therefore be potentially used as a case in point for modeling other subsystems of increasing complexity, and ultimately, the entire cell.

Using the OPCAT modeling environment (downloadable from www.opcat.com/downloads/restricted), the first step in an OPM-based modeling process is to determine and phrase the name of the main process being modeled. For example, for our case study, the main process was mRNA Lifecycle. For an entire organism, this top-level process would be Living. In an OPM-based model, the major process, such as mRNA Lifecycle, is depicted as an ellipse at the center of the System Diagram, the top level, most abstract OPD. Around this process we arrange the objects that take part in the major process, both the systemic and the environmental ones. For Living, one might put the Organism or Eukaryotic Cell as the main systemic object and substances that are needed to support life, like Oxygen, Water and Food, as the environmental objects. The Organism would be linked to Living with an effect link, denoting that Living affects Organism, while substances would be consumed and generated by the Living process. As the model is constructed graphically, sentences start showing up in the OPL text window, which the modelers in the modeling teams should read frequently to make sure that OPCAT “understands” the modelers' intentions, and correct the model in case of misinterpretations. Having obtained this top-level view of the system's structure and behavior, the Living process can be in-zoomed in a new diagram, to reveal a small number of major subprocesses, such as Breathing, Digesting, Moving, and Multiplying. In parallel, one or more objects from the System Diagram can be unfolded to reveal their parts, which can be linked to one or more of the Living subprocesses. This practice of zooming into processes while unfolding related, same level objects, can be repeated to create a hierarchical tree of Object-Process Diagrams that are fully aware of each other, since they were all created from the same System Diagram root, and each one of them focuses on some portion of combined structure and behavior of the same system at various levels of granularity. OPCAT, the OPM-based conceptual modeling environment, ensures that all the OPDs are consistent with each other in spite of the fact that they are at different detail levels. This hierarchical model structure balances the need for clarity, i.e., the need to provide comprehensible diagrams, on one hand and completeness, i.e., the need to provide as many details as possible about the objects in the system and the processes that transform them, on the other hand. Once an initial model has been designed, it can be animated to visualize the timings of process occurrences and associated object transformations.

## Discussion

In this work, we have applied Object-Process Methodology to create a conceptual model of the mRNA lifecycle. This relatively simple model demonstrates the usefulness of conceptual modeling not just for understanding and communicating the structure and behavior of cell-level systems, but also for provoking conjectures and triggering ideas for experiments that can confirm or refute such conjectures. The findings update the model, which keeps evolving by repeating this cycle. Our model includes basic, well-known aspects of the mRNA lifecycle as well as recently discovered features. We modeled deliberately both established and less established knowledge in order to demonstrate that in both cases the model generates useful predictions. The relatively new feature of the mRNA lifecycle that we focused on here is the capacity of mRNA-protein (RNP) molecules to bundle together and form large cytoplasmic complexes, termed P bodies. We hypothesized that the purported regulation of P body biology is governed by D-Factor (which may be composed of several distinct components). This hypothetical D-Factor controls the fate of mRNA by “deciding” whether each mRNA is degraded, transported to the ribosome for reuse, or sequestered in the P body.

Recently, we proposed that P bodies are heterogeneous complexes [58] and that specific P bodies interact with a particular class of mRNAs, which encode proteins sharing a common biological function (e.g., the protein biosynthetic machinery). We proposed that P bodies specialize to coordinate the regulation (storage, translation, or degradation) of classes of mRNAs. We also speculated that, in addition to their being hubs, P bodies may contribute also to the transport of mRNAs to specific locations within the cytoplasm. Such transport might be vital for large cells, e.g., nerve or dendrite cells. A recent description of mRNA localization in dendritic cells can be found in [65]. In particular, we demonstrated here that modeling recent data helps pinpoint uncertainties, raise new questions, and experiment to get answers to these questions. This is how we obtained the new result we report in this work, which establishes the localization of eRF3 in P bodies.

**Figure 7:**
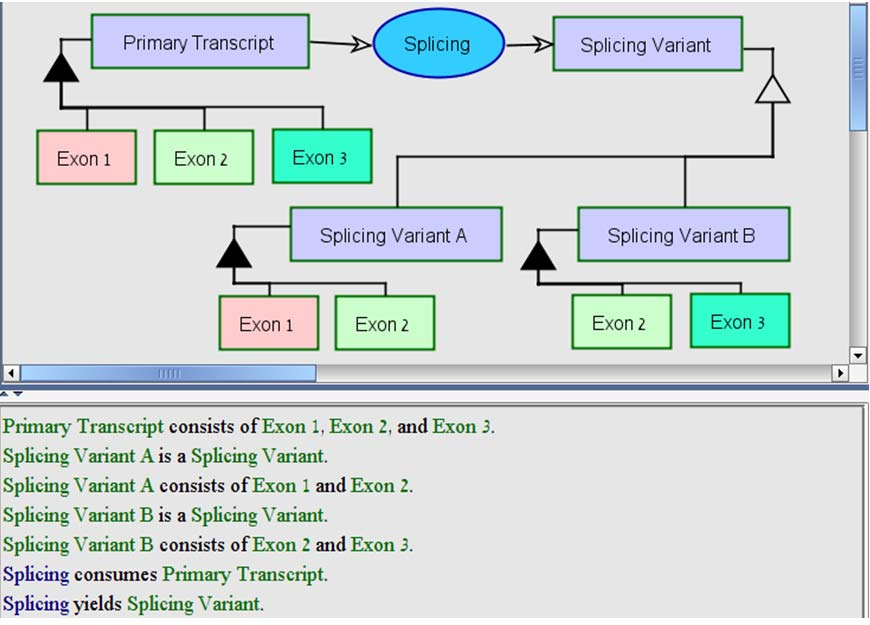
An example of modeling variants with OPM: A primary transcript is spliced to yield two splicing variants, A and B, each containing a different exon combination, as expressed in both the OPD at the top and the OPL text at the bottom. Modeling other types of variants, e.g., protein phosphorylation, protein ubiquitination, RNA editing, can be done in a similar manner.

OPM has rich and flexible modeling capabilities with high expressive power. For example, as Figure 7 shows, variants, such as splicing variants, can be modeled explicitly and clearly.

We emphasize that the OPM conceptual model of the mRNA lifecycle presented in this work is by no means complete. It illustrates superficially certain parts of this extremely complex cycle (which can be referred to as a pathway, if nucleotide recycling is ignored) as we conceive it today. Still, our model was detailed enough to provoke research questions, and a solution for one of those questions was found experimentally. The mRNA lifecycle is studied extensively, so any sections of this model can be in-zoomed further to provide ever more detailed descriptions.

In general, systems biology can benefit from using OPM as a generic framework for knowledge capture and representation, particularly since it enables balanced and unified representation of the system's structure and behavior using both objects and processes in the same diagram. Using the refinement-abstraction mechanisms that are built into OPM, the system under study can be clearly understood and communicated at various detail (granularity) levels. Moreover, as demonstrated here, OPM-based modeling provokes consideration of links missing from the process chain and stimulates ideas for experiments to prove or disprove new theories. Without the intellectual activity underlying conceptual modeling, such gaps or inconsistencies in the model can easily go unnoticed, evading the researchers' attention. Indeed, while engaged in modeling, we encountered portions of the system which we were uncertain how to model. The knowledge gaps become more apparent as we tried to further zoom (drill down) into specific subprocesses of the mRNA lifecycle. Graphically, this was manifested by increasing red color in the diagrams. Based on our positive experience, we propose that the friendly, yet formal, OPM modeling framework is a tool for modeling biological systems, whose adoption would benefit the emerging domain of systems biology.

Due to the ability to forge a holistic conceptual model of a biological system, we maintain that this research should be valuable to biologists and computer scientists who work on developing a Systems Biology understanding of the living organisms. The OPM-based conceptual modeling framework provides a clear, unambiguous way to describe our current knowledge of the state of the cell. It allows incremental resolution of different parts of the cellular machinery, helps pinpoint areas where our current understanding of the model is lacking, and finally, what type of experiments we may conduct to improve our understanding.

Following intensive future research and development, we envision an evolving model that would be developed, maintained, and updated constantly by the research community at large. When incorporating new data into the existing model, it would be possible to determine if it is consistent with previous knowledge. Unproven conjectures could also be incorporated into this evolving model, tagged as uncertain. Their inclusion in the model would help point towards evidence required to support these hypotheses. Such a modeling framework would help researchers tackling the huge challenge of understanding holistically the intricacies of the living cell.

## Supporting Information

Table S1 A Quick Guide to the Syntax and Semantics of the Object-Process Methodology (OPM) Language.
(1.06 MB DOC)
