# Supplementary material for: Conceptual Modeling in Systems Biology Fosters Empirical Findings: The mRNA Lifecycle
Source: PLoS One. 2007 Sep 12;2(9):e872. doi: 10.1371/journal.pone.0000872 (PMC1964809; doi:10.1371/journal.pone.0000872)
Supplement: Table S1 — A Quick Guide to the Syntax and Semantics of the Object-Process Methodology (OPM) Language. (1.06 MB DOC) [file pone.0000872.s001.doc]

A Quick Guide to the Syntax and Semantics of the

Object-Process Methodology (OPM) Language

Supplement 1 to

"Conceptual Modeling in Systems Biology Fosters Empirical Findings: The mRNA Lifecycle"

by Dov Dori and Mordechai Choder

[**ENTITIES**](#_ENTITIES) **
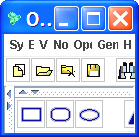
**

[**STRUCTURAL LINKS & COMPLEXITY MANAGEMENT
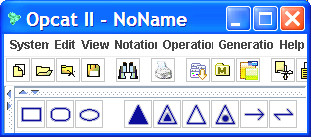
**](#_STRUCTURAL_LINKS)

[**ENABLING AND TRANSFORMING PROCEDURAL LINKS**](#_ENABLING_AND_TRANSFORMING_PROCEDURA)
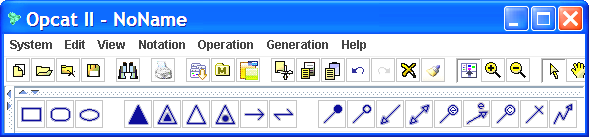


[**EVENT, CONDITION, AND INVOCATION PROCEDURAL LINKS**](#_EVENT,_CONDITION,_AND_INVOCATION_PR)
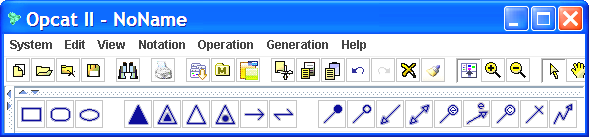

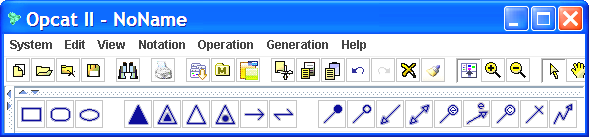


| ENTITIES | | | | **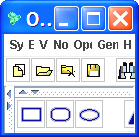** |
| --- | --- | --- | --- | --- |
| **Name** | | **Symbol** | **OPL** | **Definition** |
| Things | **Object**  **Process** | 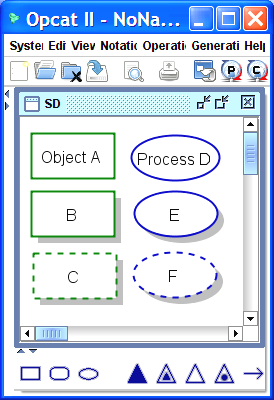 | **B** is physical.  (shaded rectangle)  **C** is physical and environmental.  (shaded dashed rectangle)  **E** is physical.  (shaded ellipse)  **F** is physical and environmental.  (shaded dashed ellipse) | An **object** is a thing that exists.    A **process** is a thing that transforms at least one object.  Transformation is object generation or consumption, or effect—a change in the state of an object. |
| **State** | | **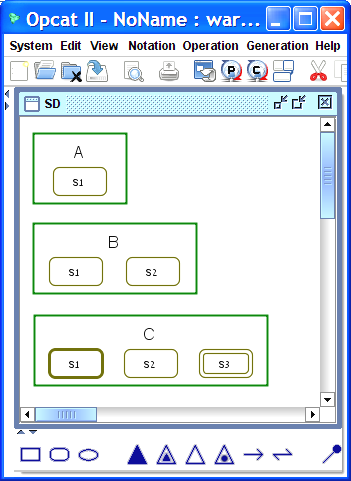** | **A** is **s1**.  **B** can be **s1** or **s2**.  **C** can be **s1**, **s2**, or **s3**.  **s1** is initial.  **s3** is final. | A **state** is situation an object can be at or a value it can assume.  States are always within an object.  States can be initial or final. |

| STRUCTURAL LINKS & COMPLEXITY MANAGEMENT | | | | 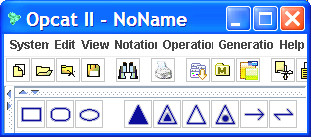 |
| --- | --- | --- | --- | --- |
| **Name** | | **Symbol** | **OPL** | **Semantics** |
| Fundamental Structural Relations | Aggregation-Participation | 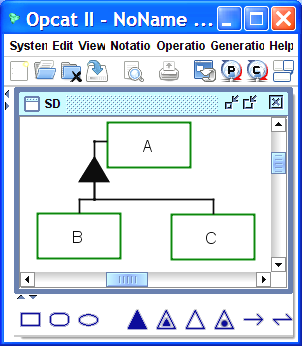 | **A** consists of **B** and **C**. | A is the whole, B and C are parts. |
|  |  | 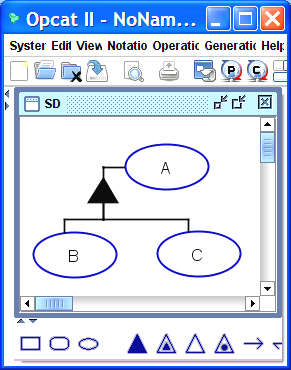 | **A** consists of **B** and **C**. |  |
|  | Exhibition- Characterization | 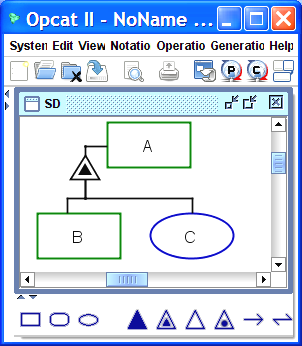 | **A** exhibits **B**, as well as **C**. | Object B is an attribute of A and process C is its operation (method).  A can be an object or a process. |
|  |  | 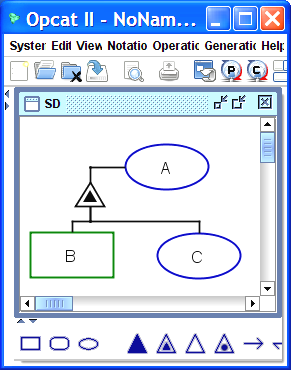 | **A** exhibits **B**, as well as **C**. |  |
|  | Generalization- Specialization | 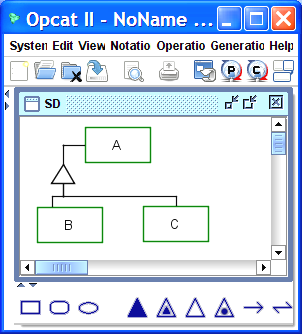 | **B** is an **A**.  **C** is an **A**. | A specializes into B and C.  A, B, and C can be either all objects or all processes. |
|  |  | 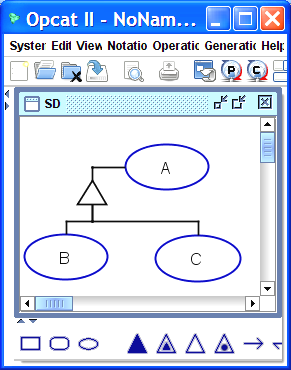 | **B** is **A**.  **C** is **A**. |  |
|  | Classification-Instantiation | 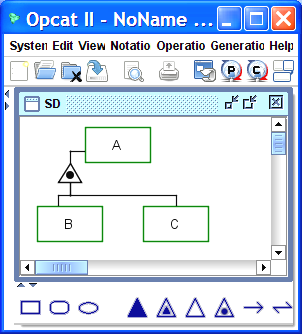 | **B** is an instance of **A**.  **C** is an instance of **A**. | Object A is the class, for which B and C are instances.  Applicable to processes too. |
| Unidirectional & bidirectional  tagged structural links | | 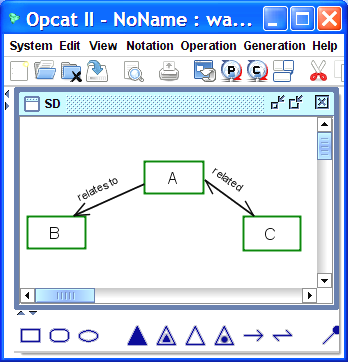 | **A** relates to **B**.  (for unidirectional)  **A** and **C** are related.  (for bidirectional) | A user-defined textual tag describes any structural relation between two objects or between two processes. |
| In-zooming | | 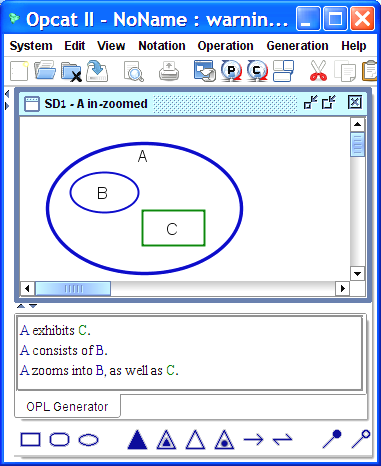 | **A** exhibits **C**. **A** consists of **B**. **A** zooms into **B**, as well as **C**. | Zooming into process A, B is its part and C is its attribute. |
|  |  | 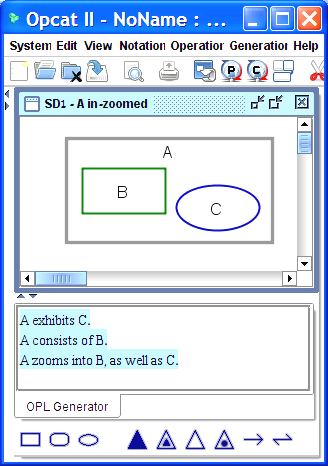 | **A** exhibits **C**. **A** consists of **B**. **A** zooms into **B**, as well as **C**. | Zooming into object A, B is its part and C is its operation. |

| ENABLING AND TRANSFORMING PROCEDURAL LINKS | | | | 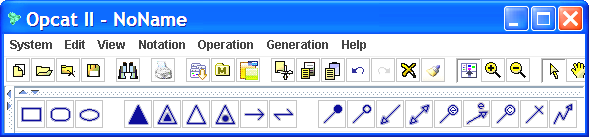 |
| --- | --- | --- | --- | --- |
| **Name** | | **Symbol** | **OPL** | **Semantics** |
| Enabling links | Agent Link | 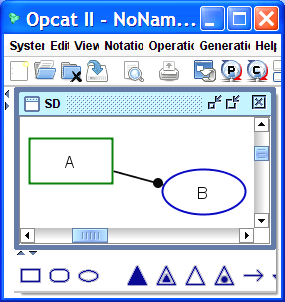 | **A** handles **B**. | Denotes that the object is a human operator. |
|  | Instrument Link | 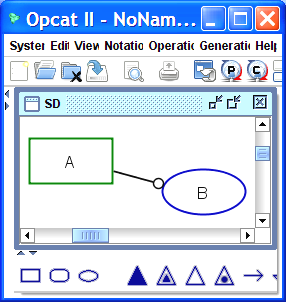 | **B** requires **A**. | "Wait until" semantics: Process B cannot happen if object A does not exist. |
|  | State-Specified  Instrument Link | 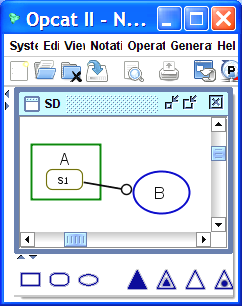 | **B** requires **s1 A**. | "Wait until" semantics: Process B cannot happen if object A is not at state s1. |
| Transforming links | Consumption Link | 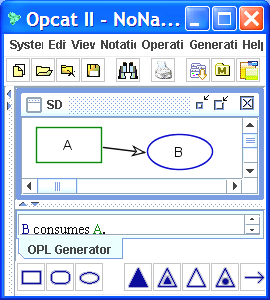 | **B** consumes **A.** | Process B consumes Object A. |
|  | State-Specified  Consumption Link | 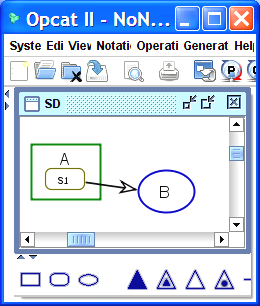 | **B** consumes **s1** **A.** | Process B consumes Object A when it is at State s1. |
|  | Result Link | 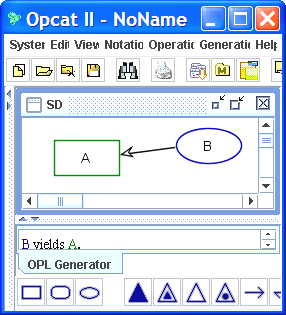 | **B** yields **A**. | Process B creates Object A. |
|  | State-Specified Result Link | 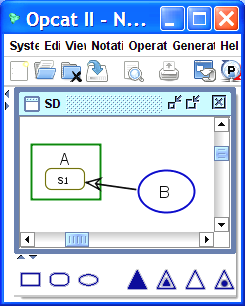 | **B** yields **s1** **A**. | Process B creates Object A at State s1. |
|  | Input-Output Link Pair | 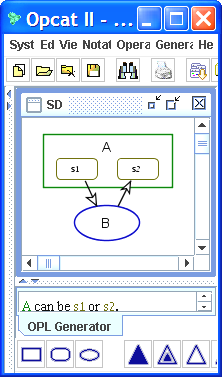 | **B** changes **A** from **s1** to **s2**. | Process B changes the state of Object A from State s1 to State s2. |
|  | Effect Link | 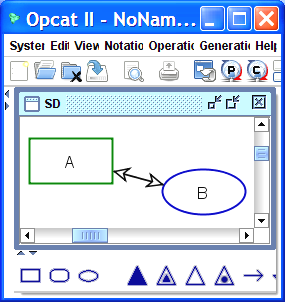 | **B** affects **A**. | Process B changes the state of Object A; the details of the effect may be added at a lower level. |

| EVENT, CONDITION, AND INVOCATION PROCEDURAL LINKS | | | 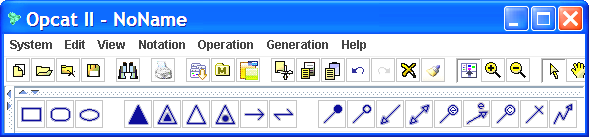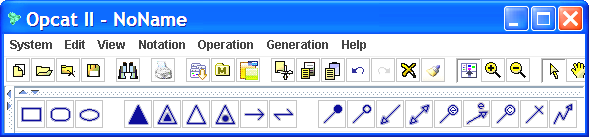 |
| --- | --- | --- | --- |
| **Name** | **Symbol** | **OPL** | **Semantics** |
| Instrument  Event Link | 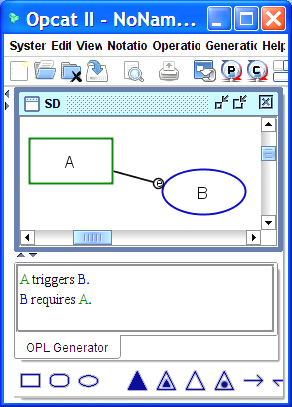 | **A** triggers **B**.  **B** triggers **A**. | Existence or generation of object A will attempt to trigger process B once. Execution will proceed if the triggering failed. |
| State-Specified  Instrument Event Link | 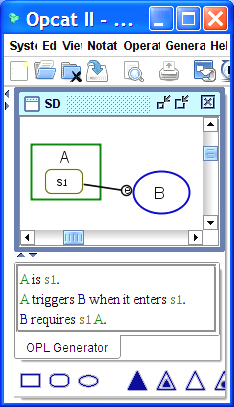 | A triggers **B**. when it enters **s1**. **B** requires **s1 A**. | Entering state s1 will attempt to trigger the process once. Execution will proceed if the triggering failed. |
| Consumption  Event Link | 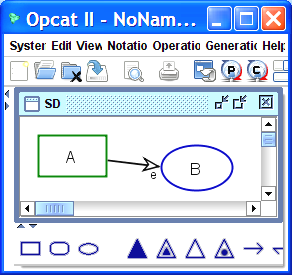 | **A** triggers **B**.  **B** consumes **A**. | Existence or generation of object A will attempt to trigger process B once. If B is triggered, it will consume A. Execution will proceed if the triggering failed. |
| State-Specified  Consumption Event Link | 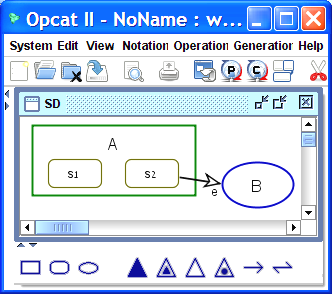 | **A** triggers **B** when it enters **s2**.  **B** consumes **s2 A**. | Entering state s2 will attempt to trigger the process once. If B is triggered, it will consume A. Execution will proceed if the triggering failed. |
| Condition Link | 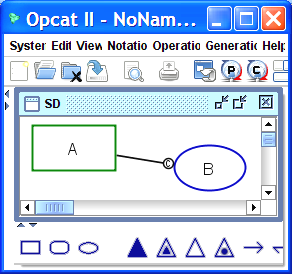 | **B** occurs if **A** exists. | Existence of object A is a condition to the execution of B.  If object A does not exist, then process B is skipped and regular system flow continues. |
| State-Specified  Condition Link | 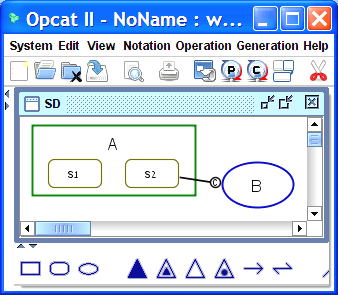 | **B** occurs if **A** is **s1**. | Existence of object A at state s2 is a condition to the execution of B.  If object A does not exist, then process B is skipped and regular system flow continues. |
| Invocation Link | 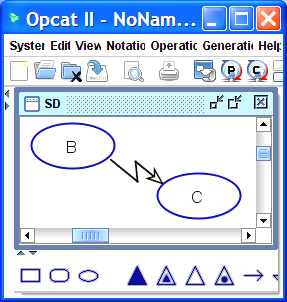 | **B** invokes **C**. | Execution will proceed if the triggering failed (due to failure to fulfill one or more of the conditions in the precondition set). |
